# Synthesis and Characterization of Silica Obtained by Combined Acid–Alkali Treatment of Serpentinite

**DOI:** 10.3390/molecules30204076

**Published:** 2025-10-14

**Authors:** Abdrazakh Auyeshov, Kazhmukhan Arynov, Chaizada Yeskibayeva, Aitkul Ibrayeva, Elmira Dzholdasova

**Affiliations:** 1 Scientific Research Laboratory “Applied Chemistry”, M. Auezov South Kazakhstan University, Tauke-Khan St. 5, Shymkent 160012, Kazakhstan; 2 LLP “Institute of Innovative Research and Technology”, 8th Microdistrict, Building 28, Apt. 2, Almaty 050010, Kazakhstan

**Keywords:** serpentinite, acid–alkali processing, amorphous silica, industrial waste utilization, magnesium hydrosilicates

## Abstract

Serpentinite rocks and their processing waste represent a valuable source of magnesium and silicon; however, their complex composition complicates the efficient recovery of individual components. This study investigates the combined acid–alkali processing of serpentinite waste from the Zhitikara deposit (Kazakhstan). In the acid leaching stage, sulfuric acid enables magnesium extraction, while subsequent treatment with sodium hydroxide (NaOH) facilitates the selective recovery of silica gel formed during acid attack. At the final neutralization step, amorphous silica is precipitated with a yield exceeding 60% of its initial content. The obtained silica was characterized using FTIR spectroscopy, X-ray diffraction (XRD), scanning electron microscopy with energy-dispersive spectroscopy (SEM-EDS), inductively coupled plasma mass spectrometry (ICP-MS, Thermo iCAP-Q), and nitrogen adsorption measurements via the BET method. It was established that the synthesized silica gel, according to the IUPAC classification, belongs to mesoporous materials, possesses a well-developed specific surface area (400 m^2^·g^−1^), and is suitable for adsorption and catalytic applications.

## 1. Introduction

Serpentinite rocks and their processing waste represent a valuable source of magnesium (up to 43 wt.% MgO) and silica (up to 45 wt.% SiO_2_). They have long attracted the attention of researchers as a potential raw material for the production of industrially important magnesium- and silica-based compounds. However, despite extensive research into the integrated processing of serpentinite, its practical implementation remains limited. This is primarily due to both technological and economic constraints that hinder the development and application of efficient recovery methods.

From a technological perspective, the challenge lies in the structural and chemical complexity of serpentinite. These rocks are mainly composed of minerals from the serpentine group—such as chrysotile, antigorite, and lizardite—with the general formula Mg_3_Si_2_O_5_(OH)_4_ or [Mg(OH)_2_(MgOH)_2_Si_2_O_5_]. While the magnesium-bearing phases are readily soluble in acids, they are unreactive in alkaline media. Consequently, most research has focused on acid-based leaching methods [1,2,3,4,5,6]. However, although these methods offer certain advantages, they also present serious limitations from both technological and environmental standpoints.

One of the major issues associated with acid leaching (e.g., using H_2_SO_4_, HCl, or HNO_3_) is the formation of insoluble polysilicic acids [7], which convert into hydrated silica gel (SiO_2_·nH_2_O) during the leaching process. This gel layer forms on the surface of the particles, impeding further dissolution, complicating solid–liquid separation, and reducing the extraction efficiency of magnesium. In particular, it severely affects filtration and the removal of metal impurities from the pregnant solutions. Extracting high amounts of magnesium from acid-resistant serpentinite (80–90%) generally requires intensified conditions—namely, leaching for ≥1 h at 70–90 °C in concentrated mineral acids (H_2_SO_4_, HCl, HNO_3_) [2,8]. Various technologies have been proposed to optimize the acid leaching of magnesium, aimed at improving process conditions as well as enhancing the technological and economic performance of acid-based methods. In particular, the amount of aggressive reagent consumed during leaching can be reduced if serpentinites are subjected to preliminary thermal treatment [9]. This approach to acid processing facilitates the dissolution of serpentine, ensuring higher yields of magnesium compounds within shorter leaching times under mildly acidic conditions. Thermal activation of serpentinites also makes it possible to reveal structural features inherent to the silicate layers of their polymeric structure, as well as the characteristics of their thermal decomposition accompanied by the formation of a certain amount of free MgO phases in the system [10]. However, concerns have been raised that the beneficial effects of thermal activation are likely to be offset by increased production and energy costs of magnesium products derived from serpentinite. In order to overcome these shortcomings, the use of low concentrations of acids (up to 30–40% of the stoichiometric amount of H_2_SO_4_ required) has been proposed, under which the amount of SiO_2_·nH_2_O gel formed is insufficient to block the surface of serpentinite particles. This method makes it possible to achieve a high acid utilization coefficient (up to 98–100%), while the degree of magnesium extraction amounts to 30–40% [11]. The technological and economic aspects of developing processing technologies for serpentinites and the large-tonnage chrysotile waste accumulated worldwide remain highly relevant. Therefore, further research on serpentine dissolution is required to develop alternative approaches to serpentinite processing that can address the existing technological, economic, and environmental challenges. In the present study, another approach to acid processing of serpentinite was applied, combining acid–alkali leaching for the simultaneous recovery of magnesium and silicon. The development of this method makes it possible to minimize the above-mentioned problems. Reports on such processes in the literature are scarce and appear only sporadically. Matus et al. (2020) [12] demonstrated the effective extraction of magnesium from olivine using sequential leaching. Ulum et al. (2023) [13] developed an optimized process for obtaining high-purity silica from slags through alkaline precipitation. Other studies have shown the potential for using serpentine-based residues to produce functional materials such as adsorbents and construction additives [14]. One of the features of obtaining amorphous silica from layered magnesium silicates is that silica produced through acid treatment retains the morphology of the original minerals [15]. Typical values of the specific surface area of silica obtained from layer silicates are as follows: from talc, SBET ≈ 130 m^2^·g^−1^ [16]; from olivine/serpentinites, SBET up to 1000 m^2^·g^−1^ [17,18]; and from serpentinite, SBET up to 50–400 m^2^·g^1^ [19]. Through multistage purification of serpentinite, high-purity SiO_2_ (>99%) can be obtained while preserving the microstructure. The adsorption properties of amorphous silica derived from layered magnesium silicates largely depend on the morphology of the serpentinite raw material (from different deposits). The morphology of the resulting silica is also influenced by the mechanism of gel formation and evolution under acid treatment (removal of magnesium → release of H_4_SiO_4_, and/or formation of silica-rich layers). Acids ([H^+^]) simultaneously accelerate the dissolution of magnesium and modify the rates of polymerization and flocculation of silica. In some cases, the reaction rate at the initial stage approximates first-order kinetics with respect to [H_2_SO_4_]. Consequently, the rate constant increases with increasing [H^+^]. In turn, the rate of polycondensation of monosilicic acid is also a function of the pH of the medium. Thus, under different acid treatment conditions, various types of amorphous silica with different pore structures and parameters may be formed, which define its functional applicability in diverse fields. Therefore, it is necessary in each case to investigate their adsorption properties in order to identify potential areas of application. The search for new approaches and alternative pathways for the utilization of serpentinite to obtain valuable products is highly relevant for Kazakhstan, which possesses significant reserves of serpentinite rocks and large volumes of chrysotile asbestos waste. In the context of increasing interest in technologies for processing industrial waste, this approach may serve as a basis for the production of useful magnesium- and silica-based products from serpentinite tailings in Kazakhstan and in other countries rich in chrysotile raw materials.

The aim of this study is to investigate the processes of combined acid–alkali treatment of serpentinite waste from the Zhitikara deposit (Kazakhstan). Physicochemical analysis methods (XRD, FTIR spectroscopy, BET adsorption) were employed to study the mechanisms of serpentinite dissolution in sulfuric acid, as well as the adsorption characteristics of amorphous silica obtained through acid and combined acid–alkali treatment of serpentinite.

## 2. Results and Discussion

The combined method involving acid and alkali treatment is based on sequential processes occurring during the dissolution of serpentinite in acid and subsequent treatment of the acid-insoluble residues in alkaline media:

During the acid leaching stage, the destruction of the crystalline lattice of serpentinite occurs, particularly in the regions of Mg–OH bonds and partially in Si–O–Mg bonds:

Mg(OH)_2_(MgOH)_2_Si_2_O_5_ + 6H^+^ → 3Mg^2+^ + 2SiO_2_ + 5H_2_O (1)

Mg^2+^ ions enter the solution, while the formed amorphous silica (SiO_2_·nH_2_O) creates a gel-like phase, which causes technological complications during the filtration and purification stages.

The amorphous SiO_2_·nH_2_O reacts well with alkalis, transforming into soluble silicates:

SiO_2_·nH_2_O + 2NaOH → Na_2_SiO_3_ + (n + 1) H_2_O (2)

### 2.1. Interactions in the “Serpentinite–H_2_SO_4_” System

The acid treatment of the initial serpentinite waste (SP^0^) was performed following the procedure outlined in the Section 3. Based on chemical analysis, the compositions of the filtrate and the insoluble residue were determined, along with the leaching efficiencies of individual elements from SP^0^. The results are summarized in Table 1.

According to Table 1, during the initial acid leaching of the serpentine waste SP^0^ with sulfuric acid solution, a significant portion of magnesium, manganese, chromium, calcium, and aluminum (over 50%) is transferred into the sulfate solution.

**Figure 1:**
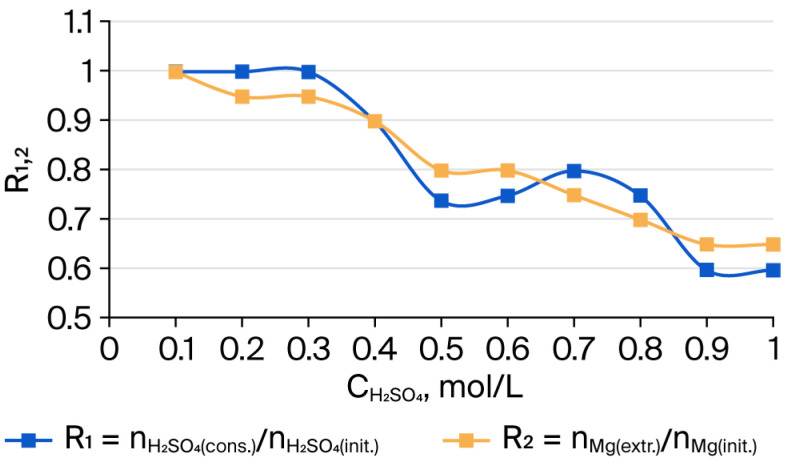
Dependence of the molar ratios R_1_ = $n_{H_{2}{SO}_{4}(cons.)}/n_{H_{2}{SO}_{4}(init.)}$ and R_2_ = $n_{Mg(extr.)}/n_{Mg(init.)}\;$ on the treatment of 10 g of serpentinite with sulfuric acid solutions (0.1–1.0 mol/L), corresponding to the stoichiometrically required amount, and with τ = 10 min and t = 98 °C.

**Figure 2:**
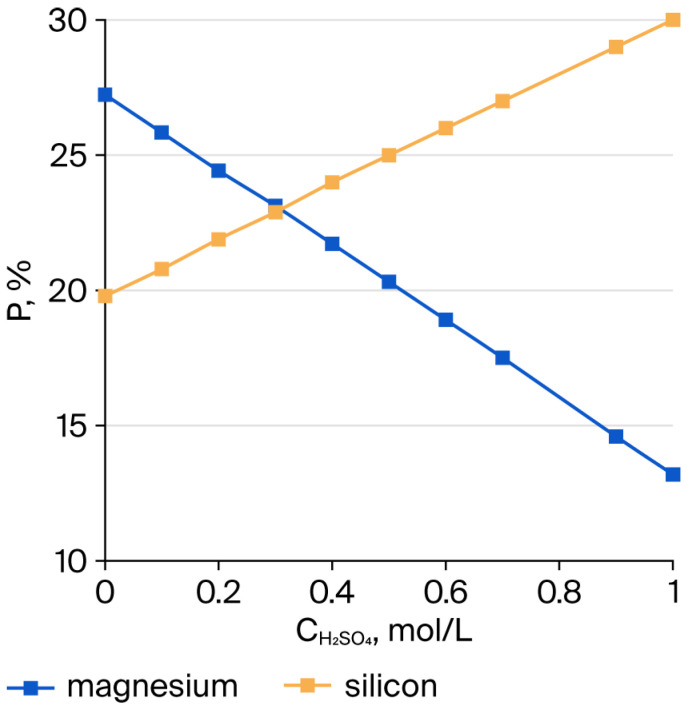
Variation in magnesium and silicon concentrations in the insoluble residue after the interaction of serpentinite with H_2_SO_4_, at a ratio of 10 g of serpentinite Mg (1.1 mol/L)/H_2_SO_4_ (0.1–1.0 mol/L), using the stoichiometrically required amount, for τ = 10 min and t = 98 °C.

**Figure 3:**
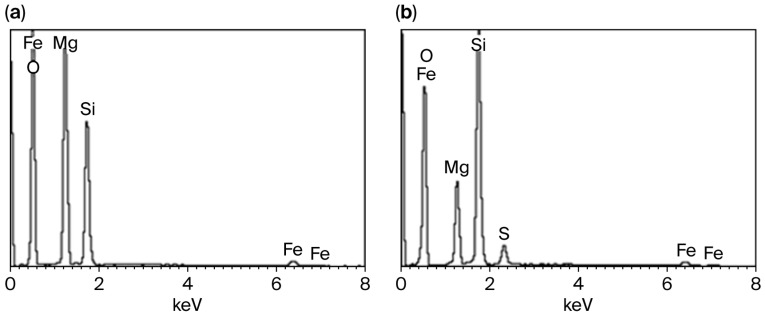
Energy-dispersive spectra of the initial serpentinite (**a**) and after treatment with H_2_SO_4_ solution at SP:H_2_SO_4_ = 1:1 (stoichiometrically required amount) (**b**).

At the same time, iron was leached only weakly (27%), and silicon to an insignificant extent (2%). The insoluble residue after leaching was substantially enriched with silicon (up to 98%) and iron (up to 73%). The general trend of changes in the composition of acid-insoluble residues depending on the concentration of H_2_SO_4_ used is shown in Figure 1, Figure 2 and Figure 3. Figure 1 illustrates the consumption of sulfuric acid and the change in the amount of Mg extracted from serpentinite (SP). As can be seen, when SP (Mg) and H_2_SO_4_ are taken at a molar ratio of 1:1, the maximum consumption of H_2_SO_4_ for Mg extraction is about 0.6, and the amount of acid not involved in the reaction is comparable to the value of free acid in the solution after the interaction, as determined through titration with NaOH.

The observed fact that a certain amount of free H^+^ remains in the solution indicates incomplete progress of the reaction of SP with sulfuric acid.

The dissolution of magnesium from the crystalline framework of SP as a result of its interaction with H^+^ ions leads to the formation of silicon dioxide accompanied by the complete destruction of the molecular structure of the outer layers of SP fibers, which consist of tubes coiled into bundles. The dissolution of these tubular layers, in general, appears to proceed in a predominantly stepwise manner. As shown in Figure 1, the variations of $C_{H_{2}{SO}_{4}(cons.)}/C_{H_{2}{SO}_{4}\left(init.\right)}$ and $C_{{Mg}^{2+}(extr.)}/C_{{Mg}^{2+}\left(SP\right)\;}$ exhibit approximately the same trend. The changes in the amounts of Si and Mg in the insoluble residue after treatment of serpentinite with H_2_SO_4_ are presented in Figure 2. The simultaneous decrease in Mg content and increase in Si content in the residue indicate that the interaction of sulfuric acid with SP proceeds in a predominantly stepwise fashion.

Comparative energy-dispersive spectra of SP before and after treatment with 1.0 mol/L H_2_SO_4_ (Figure 3) show that the Mg content in the residue decreases by approximately 60–65%. The residue still contains about 12% magnesium, which is most likely only located in the unreacted inner layers of the SP fibers.

The unreacted layer-packages retain their molecular–structural framework, which is protected from further destruction by a denser hydrated silica surface layer that limits the subsequent diffusion of H^+^ ions into the deeper parts of the SP particles.

### 2.2. XRD and FTIR Spectroscopy

**Figure 4:**
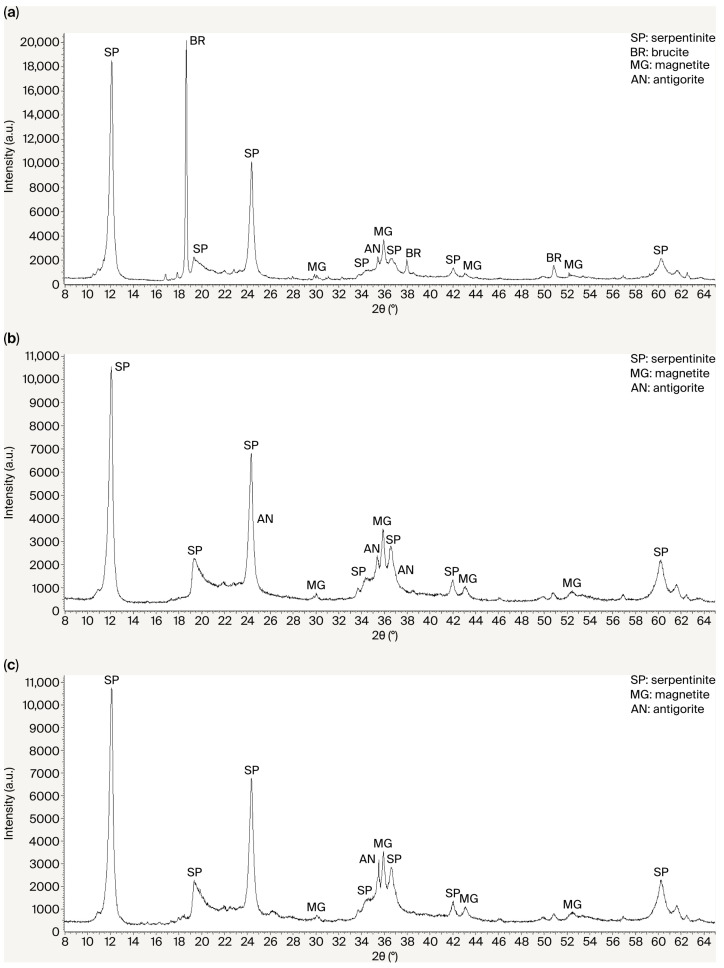
X-ray diffraction patterns of the initial serpentine waste SP^0^ (**a**), the acid-insoluble residue SP^I^ (**b**), and the alkali-insoluble residue SP^II^ (**c**).

Phase transformations occurring during the acid–alkali treatment of the initial serpentinite are shown in Figure 4a–c. In the XRD pattern of the raw serpentinite (a), the identified phases are chrysotile (SP), antigorite (AN) Mg_3_Si_2_O_5_(OH)_4_, and magnetite Fe_3_O_4_. In the XRD pattern of the acid-treated serpentinite SP^I^ (b), the identified phases are chrysotile (SP), antigorite (AN) Mg_3_Si_2_O_5_(OH)_4_, and magnetite Fe_3_O_4_. The main changes in the phase composition after acid treatment include the disappearance of brucite [Mg(OH)_2_] (BR) reflections at interplanar spacings of d/n = 4.77, 2.365, and 1.794 Å. The phase transformation associated with the formation of amorphous silica during sulfuric acid treatment of serpentinite is not manifested in the XRD pattern of the acid-insoluble residue. However, the twofold decrease in the intensity of the main serpentinite (SP) peaks at d/n = 7.38, 3.661, 2.487, and 1.53 Å may be attributed to the formation of an amorphous Si-rich layer on the surface of the serpentinite particles.

During the alkaline treatment of the acid-insoluble residue (SP^I^) (Figure 4c), the characteristic X-ray diffraction reflections of the serpentinite minerals (SP^0^) reappeared with their initial intensity. The original texture of serpentinite was restored. Upon repeated treatment (additional verification) of the alkaline residue of the acid-treated serpentinite (SP^III^), the formation of hydrated amorphous silica was again recorded, along with SiO_2_·nH_2_O phases. The serpentinite peaks regained their previous intensity. The changes observed in the diffraction patterns after the acid–alkali treatment of serpentinite demonstrate that acid exposure resulted in the formation of a multilayer silica coating enriched with magnesium disilicate. The acid simultaneously accelerated magnesium dissolution and modified the rate of silica polymerization and flocculation. As a result, a gel/Si-rich layer was formed.

**Figure 5:**
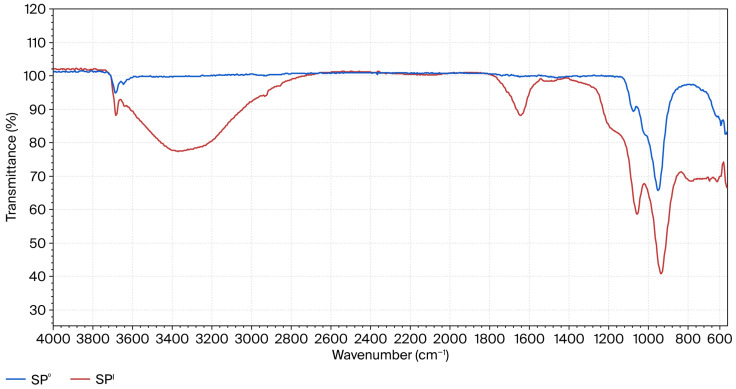
FTIR spectra of the initial sample SP^0^ and the acid-insoluble residue SP^I^ after treatment [7].

The mechanism of the transformation of silicate fragments of serpentinite into amorphous silica during acid treatment has been reported previously [7]. Specific characteristic absorption bands of amorphous silica (νas(Si–O–Si)) and hydroxyl groups (Si–OH) were detected in the FTIR spectra (Figure 5) of acid-treated serpentinite samples after treatment with H_2_SO_4_ solutions at concentrations close to C = 0.7–1.0 M (stoichiometrically required amount of H_2_SO_4_). These absorption bands simultaneously indicated a significant disruption of the tetrahedral–octahedral connectivity of the crystalline lattice of serpentinite. At the same time, the presence of vibrations associated with iron was not detected in the FTIR spectrum. Apparently, during dissolution, the isomorphically incorporated iron atoms do not participate in the formation of the Si-rich layer.

Broad intense peaks at 1160–2040 cm^−1^ indicate the presence of various acidic silanol (–Si–OH) groups, while the wide band at 3600–3200 cm^−1^ is associated with the formation of different siloxane (–Si–O–Si–OH) and hydrated groups of silica monomers, dimers, and tri- and tetra-polymers, as well as branched silica networks on the surface of serpentinite particles, marking the onset of silica gel formation on the surface layer of SiO_2_·nH_2_O [20].

The results obtained from experimental XRD and FTIR studies suggest the possibility of extracting amorphous silica formed in the SP–H_2_SO_4_ system as a separate product by applying the combined acid–alkali treatment method.

### 2.3. Production of Amorphous Silica via the Combined Acid–Alkali Method and Its Properties

According to the elemental analysis of the acid-insoluble residue (SP^I^) obtained after leaching the initial sample SP^0^ with sulfuric acid solution, the following mass fractions were determined: Mg—15.0%; Si—21.2%; Fe—3.0%; Cr—0.19%; and S—3.18%. Calculations based on the magnesium, silicon, and oxygen contents indicated that the composition approximately corresponds to the formula 1.25 MgO·1.5 SiO_2_·3H_2_O [SP^I^]. The insoluble residue weighing 83.6 g (SP^I^ obtained after acid treatment) was subjected to alkaline treatment (stage 2). Calculations performed to establish the stoichiometry of the interaction of this composition with NaOH solution and the conditions for silica precipitation were carried out according to Reactions (1) and (2), presented in Table 2.

According to Reaction (1) (Table 2), 325 cm^3^ of 4.0 M NaOH solution (ρ = 1.155 g/cm^3^) was used for the treatment.

Elemental analysis of the initial sample (SP^0^), the acid-leached residue (SP^I^), and the alkaline residue (SP^II^) showed that after the two-step treatment SP^0^ (acid and alkaline), the final residue SP^II^ became comparable in composition to the original SP^0^ in terms of the main elements (Mg and Si). In SP^0^, the molar Mg/Si ratio was 1.68, while in SP^II^ it was 1.56. These data indicate that after alkaline treatment, i.e., the removal of the silica layer from the surface of SP^I^ particles, the inner layers retain the serpentine structure [Mg_3_Si_2_O_5_(OH)_4_]. To confirm this assumption, the alkaline residue (SP^II^, 36 g) was subjected to repeated acid leaching using a 2.0 M H_2_SO_4_ solution (stage 3). The resulting acid-insoluble residue (SP^III^) was subsequently treated with alkali to obtain the alkaline residue (SP^IV^). The experimental procedures applied at stages 3 (H_2_SO_4_ treatment) and 4 (NaOH treatment) were analogous to those used at stages 1 and 2. The obtained SP^III^ and SP^IV^ residues were also analyzed following the same approach as for SP^I^ and SP^II^. The results of the elemental analysis of the insoluble residues (the average value of three parallel experiments) after sequential acid–alkaline treatment (H_2_SO_4_ and NaOH) are summarized in Table 3.

From the data in Table 3, it should be noted that the contents of the main elements, magnesium and silica, after alkaline treatment (stages 2 and 4) are close to those in the acid-insoluble residue of the initial serpentinite (SP^0^). In the conditional compositions of the acid-insoluble residues at various stages of acid–alkali extraction of magnesium and silica, enrichment in silica occurs, with a change in the MgO/SiO_2_ ratio in the series SP^0^ → SP^I^ → SP^III^ equal to 1.5 → 0.83 → 0.46, respectively.

The yield of amorphous silica (after two acid and two alkaline stages) is as follows: (1) 95.78% from 83.6 g of the acid-insoluble residue (SP^I^); (2) 60.3% from the initial serpentinite (SP^0^). Thus, the results demonstrate that the acid–alkali method makes it possible to process serpentinite waste with selective silicon extraction at sufficiently high yields by regulating the number of sequential acid and alkali leaching stages.

### 2.4. Study of the Properties of Amorphous Silica

The amorphous silica obtained by the combined acid–alkali method was investigated using ICP–MS (Thermo iCAP-Q), FTIR spectroscopy, and BET adsorption analysis.

**Figure 6:**
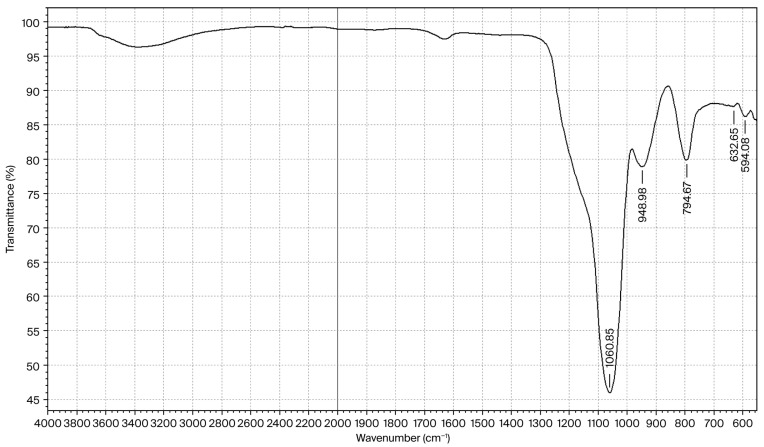
FTIR spectrum of silica with the composition SiO_2_·0.36H_2_O.

The composition of the amorphous SiO_2_ determined via ICP–MS (Thermo iCAP-Q) showed the presence of the following impurities (wt.%): Al—0.1696; Ca—0.2025; Na—0.7010; the results showed a total impurity content of 1.08%, and 8.0% adsorbed H_2_O. The formal composition corresponds to SiO_2_·0.36H_2_O. The FTIR spectrum of the amorphous silica of this composition is shown in Figure 6.

In the FTIR spectrum of silica, valence and deformation vibrations of water are observed at the following frequencies: νas(O–H)—3200–3600 cm^−1^; δ(H–O–H)—1642 cm^−1^, while asymmetric and symmetric stretching vibrations are observed for the siloxane group (νasSi–O–Si) at 1064 cm^−1^ and for νsSi–O–Si at 794 cm^−1^. Vibrations of the silanol group (νSi–OH) are observed at 948 cm^−1^, which is characteristic of amorphous silica [20].

**Figure 7:**
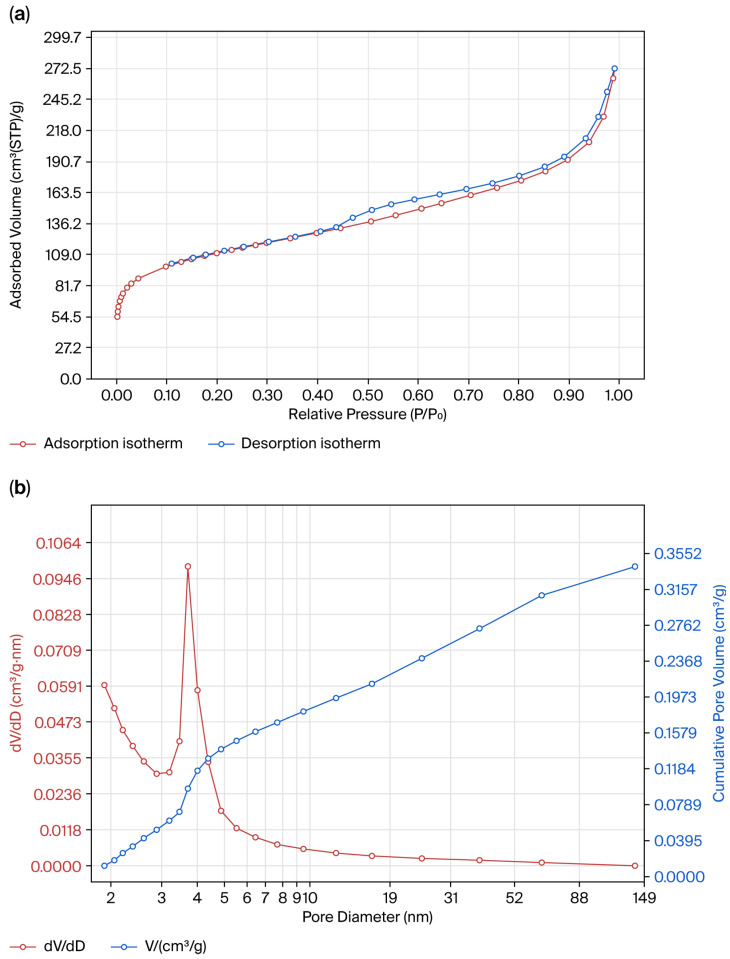
Nitrogen adsorption–desorption isotherms at 77 K (BET) (**a**) and pore size distribution (BJH) (**b**) of the synthesized amorphous silica.

The shape of the nitrogen adsorption–desorption isotherm hysteresis loop for the synthesized silica indicates a type IV isotherm with an H3 hysteresis loop (Figure 7a), which confirms the presence of a micro- and mesoporous structure with partial condensation of nitrogen in the pores of the solid. The pronounced rise in adsorption observed from 0 to 90 cm^3^·g^−1^ in the relative pressure range of P/P_0_ = 0–0.2 corresponds to the presence of micropores with diameters ranging from 0.35 to 2 nm. In addition, a small step on the desorption branch further confirms the slit-shaped nature of the pores. In the relative pressure range of P/P_0_ = 0.2–0.96, a significant increase in adsorption capacity from 90 cm^3^·g^−1^ to 204 cm^3^·g^−1^ was observed, which is characteristic of micro- and mesopores. At P/P_0_ > 0.96, the adsorption capacity increases further to 65–68 cm^3^·g^−1^, indicating the presence of macropores (50–200 nm). At the same time, in the case of this silica, a significant contribution of micro- and mesopores is evident, which is typical for microporous sorbents, as also supported by the steep rise in the adsorption curve at low relative pressures and at P/P_0_ ≈ 0.96.

The results of the pore distribution analysis conducted via the BJH method (desorption branch) (Figure 7b) revealed a pronounced peak at ~3.7 nm, indicating the predominance of mesopores and a uniform pore structure of the material. Additionally, the presence of micropores (1.7–2.1 nm), confirmed via the DR and DA methods, was observed. The pore size distribution confirmed that the obtained silica is a micro- and mesoporous material. Micropores (0.35–2 nm) accounted for 46% of the surface area and 21% of the pore volume, while mesopores (2–10 nm) comprised 48% of the surface area and 42% of the pore volume. Thus, micropores and mesopores together provided the largest contribution, 95% of the surface area and 63% of the pore volume, which supports the structural uniformity of the synthesized amorphous silica. The total pore volume of 0.42 cm^3^·g^−1^ indicates the developed porous structure, making this material promising for adsorption and catalytic applications. The main parameters characterizing the textural properties of silica are summarized in Table 4.

The analysis of the textural characteristics of silica showed that the material possesses a high specific surface area of 400 m^2^·g^−1^ (BET method) and 559.13 m^2^·g^−1^ (Langmuir method), which is consistent with the data on the developed mesoporous structure. The total pore volume was 0.42 cm^3^·g^−1^, while the contribution of micropores (T-plot) was 0.09 cm^3^·g^−1^, confirming the combined micro- and mesoporous nature of the material. The average pore diameter calculated using the BET method (4V/S) was 4.18 nm, whereas the BJH method (desorption branch) yielded larger values of 5.68 nm due to the features of mesopore distribution. The most probable pore diameter (dmode) from BJH desorption was 3.73 nm, indicating the predominance of relatively narrow slit-like mesopores.

Additional calculations by the DR and DA methods revealed micropores with diameters of 1.57–2.14 nm, and the Horvath–Kawazoe (H–K) method also confirmed the presence of micropores in the range of 1.6–1.9 nm. Thus, the material demonstrates a bimodal pore distribution, with micropores <2 nm and mesopores around 3–6 nm. The main contribution to the textural properties is from mesopores with a diameter of 3–6 nm, accounting for ~64% of the surface area and ~52% of the pore volume. The contribution of micropores (<2 nm) was ~21% of the surface area and ~46% of the pore volume. The presence of a narrow pore size distribution and the combined micro- and mesoporous structure confirm the structural uniformity of the synthesized silica. According to the IUPAC classification, the nitrogen adsorption isotherm of silica corresponds to type IV with an H3 hysteresis loop, which is characteristic of mesoporous materials with slit-shaped pores formed by aggregates of plate-like particles. The high specific surface area (400 m^2^·g^−1^) and significant total pore volume (0.42 cm^3^·g^−1^) are described in the updated IUPAC recommendations (2015) [21] as a ‘well-developed mesoporous structure’, which corresponds to industrial adsorbents (desiccants, catalyst supports, chromatography).

In this study, other parameters influencing the extraction of silica from the acid-insoluble residue (such as temperature, stoichiometry of reagents, and others) that could affect the resulting textural properties of amorphous silica were not specifically considered. However, the obtained results indicate that amorphous silica with favorable textural characteristics can be achieved through the combined acid–alkali method of serpentinite processing.

## 3. Materials and Methods

Serpentinite waste with a particle size of ≤0.14 mm from the Zhitikara deposit (Kazakhstan) was used as the raw material. The elemental composition of the sample was as follows (wt.%): Mg—25.0; Si—17.45; Fe—2.93; Al—0.54; Ca—0.50.

X-ray diffraction (XRD) patterns were recorded using a D8 Advance diffractometer (Bruker AXS GmbH, Karlsruhe, Germany) operating with Cu-Kα radiation at 40 kV and 40 mA. Diffraction data were processed using the EVA software (version 4.2) package, and phase identification was carried out using the Search/Match function based on the PDF-2 Powder Diffraction File (JCPDS-ICDD). The phases were identified according to the JCPDS database: serpentine—16-0613, Mg(OH)_2_—07-0239, MgO—45-0946.

Fourier-transform infrared (FTIR) spectra were recorded using a Shimadzu IR Prestige-21 spectrometer (Shimadzu Corporation, Kyoto, Japan) equipped with an ATR (attenuated total reflectance) Miracle accessory (Pike Technologies, Madison, WI, USA).

The specific surface area and pore size distribution were determined through the static capacity method using a BSD-660S A3 instrument (BSD Instrument Technology, Beijing, China).

Elemental distribution during leaching and purification was analyzed using a JSM-6490LV scanning electron microscope (JEOL Ltd., Tokyo, Japan) equipped with an INCA Energy 350 energy-dispersive X-ray spectroscopy (EDS) system and an inductively coupled plasma mass spectrometer (ICP-MS, Thermo iCAP-Q, Thermo Fisher Scientific, Bremen, Germany).

Amorphous silica (SiO_2_) was produced through a combined acid–alkali method. For the experiment, 100 g of serpentine waste (SP^0^) containing 25.75 g or 1.073 mol of Mg was used. According to reaction Equation (1), complete leaching of Mg from 100 g of SP^0^ requires 1.073 mol of H_2_SO_4_.

The reactor was charged with 455 cm^3^ of a solution containing 1.073 mol of H_2_SO_4_, and 100.0 g of SP^0^ was gradually added to the solution under stirring, with time monitoring initiated simultaneously. During the addition, intensive boiling was observed, caused by the exothermic reaction. The suspension was maintained under heating for 2 h, after which it was filtered hot via vacuum suction through a blue-ribbon filter. The filtration time was 2 h. The total volume of the obtained filtrate together with the wash water was 470 cm^3^, with a pH of 0.64, while the mass of the insoluble residue after drying at 105 °C was 83.6 g. The transparent pale-green filtrate represented a solution of metal sulfates—MgSO_4_, CaSO_4_, Al_2_(SO_4_)_3_, CrSO_4_, and Fe_2_(SO_4_)_3_.

To obtain amorphous silica, the acid-insoluble residue of SP^0^ was treated with alkali according to reaction (3):

1.25MgO·1.5SiO_2_·3H_2_O + NaOH → 1.25MgO·0.075SiO_2_·3H_2_O+ 1.425Na_2_SiO_3_ (3)

An amount of 83.6 g of the acid-insoluble residue was treated with 325 cm^3^ of a 4.0 M NaOH solution under stirring for 0.5 h, after which it was filtered under vacuum, and the precipitate was washed with distilled water. Filtration proceeded slowly but satisfactorily. The volume of the alkaline filtrate was V = 1000 cm^3^, with a pH of 11.46. The filtrate was neutralized with 20% H_2_SO_4_ solution to a pH of 4.5–5.0 and then left to allow silica precipitation (Table 2, reaction 2). After 12–14 h, a finely dispersed white precipitate of silica was formed. The precipitate was filtered and washed with distilled water until no SO_4_^2−^ ions were detected in the wash water.

After drying at 105 °C, the obtained silica was analyzed using ICP-MS (Thermo iCAP-Q).

## 4. Conclusions

The combined acid–alkali processing method overcomes the limitations of traditional one-stage approaches by ensuring efficient dissolution of magnesium and selective extraction of silicon from serpentinite waste. The dissolution of the surface silica-enriched layer in an alkaline medium, formed as a result of acid treatment, promotes subsequent access of H_3_O^+^ ions to the inner layers of the mineral. The obtained amorphous silica is characterized by high purity (>95% SiO_2_) and a developed mesoporous structure, which makes it a promising material for use as an adsorbent or catalyst support. The obtained results, along with further development of the combined acid–alkali method for extracting magnesium and silicon from serpentinite, may strengthen the feasibility of this approach for designing new technological schemes for serpentinite processing.

## Figures and Tables

**Table 1:** Distribution of elements in the filtrate and insoluble residue after acid leaching of the initial serpentinite waste SP^0^.

Element	Mg	Si	Fe	Mn	Cr	Ca	Al
In filtrate (wt.%)	64.8	2.0	27.0	100.0	78.0	92.0	66.0
In residue (wt.%)	35.2	98.0	73.0	–	22.0	8.0	34.0
Total	100.0	100.0	100.0	100.0	100.0	100.0	100.0

Note: Values are expressed as percentages of the initial elemental content in the serpentinite waste (SP^0^). The leaching was carried out using sulfuric acid at a concentration of 1.073 mol/L.

**Table 2:** Reactions and stoichiometric calculations for the interaction of the acid-insoluble residue (SP^I^) with NaOH for silica precipitation.

Process	Reaction	Molar Mass, g/mol	Mass, g
Alkaline treatment of SP^I^	Reaction (1): 1.25MgO·1.5SiO_2_·3H_2_O + 3NaOH → 1.5Na_2_SiO_3_ + 1.25Mg(OH)_2_ + 3.25H_2_O	194.00 + 120.00 → 183.00 + 72.5 + 58.5	83.60 + 51.70 → 78.86 + 31.24 + 25.21
Acid treatment of silicate	Reaction (2): Na_2_SiO_3_ + H_2_SO_4_ → SiO_2_↓ + Na_2_SO_4_ + H_2_O	122.00 + 98.00 → 60.00 + 142.00 + 18.00	78.86 + 63.35 → 38.78 + 91.78 + 11.64

Note: The calculations are based on the average elemental composition of the acid-insoluble residue (SP^I^) obtained after leaching with sulfuric acid. Reaction (1) represents the alkaline decomposition of hydrated magnesium silicate and formation of sodium silicate, while Reaction (2) describes the precipitation of silica from sodium silicate using sulfuric acid.

**Table 3:** Elemental composition (wt.%) of insoluble residues after sequential acid–alkali treatment of serpentinite waste (SP^0^) with H_2_SO_4_ and NaOH solutions.

Element	Initial SP^0^	Stage 1 H_2_SO_4_ (SP^I^)	Stage 2 NaOH (SP^II^)	Stage 3 H_2_SO_4_ (SP^III^)	Stage 4 NaOH (SP^IV^)
O	51.68	60.04	48.71	42.50	35.71
Mg	25.60	12.61	25.01	12.81	22.95
Al	0.47	0.19	0.27	n.d.	n.d.
Si	17.95	21.04	18.71	31.90	18.17
S	n.d.	4.85	n.d.	n.d.	n.d.
Ca	0.56	0.05	0.18	n.d.	n.d.
Cr	0.20	0.04	0.17	0.04	0.32
Mn	0.11	n.d.	n.d.	n.d.	n.d.
Fe	3.43	3.00	6.95	12.75	22.85
Σ (wt.%)	100.00	100.00	100.00	100.00	100.00
Conditional composition	3MgO·2SiO_2_ ·2H_2_O	1.25MgO·1.5SiO_2_ ·3H_2_O	1.13MgO·0.72SiO_2_ ·0.72H_2_O	0.53MgO·1.14SiO_2_ ·H_2_O	1.24MgO·0.84SiO_2_

Note: Elemental composition is given in weight percent (wt.%). n.d.—not detected. Conditional composition is estimated based on the main oxide components and assumed hydration level.

**Table 4:** Main parameters characterizing the textural properties of amorphous silica (SiO_2_).

Sample	S, m^2^·g^−1^	ΣVpore, cm^3^·g^−1^	Vmicro, cm^3^·g^−1^	davg, nm		dmode, nm	Isotherm Type	Hysteresis Loop
Amorphous Silica (SiO_2_)	400.06 (BET P/P_0_ = 0.02–0.13)	0.42 (T-Plot)	0.09 (T-Plot)	4.18 (BET)	5.68 (BJH desorption)	3.73 (BJH desorption)	IV	H3
	559.13 (Langmuir)	0.42 (Langmuir)	0.15 (DR)	1.57 (DR micro)	1.82 (DA micro)	2.14 (DA)		

Notes: S, m^2^·g^−1^—specific surface area (BET, Langmuir methods); ΣVpore, cm^3^·g^−1^—total pore volume; Vmicro, cm^3^·g^−1^—micropore contribution (T-plot, DR, H–K); davg, nm—average pore diameter (BET, DR, DA, BJH); dmode, nm—most probable pore diameter (BJH, DA).

## Data Availability

The datasets generated and/or analyzed during the current study are available from the corresponding author upon reasonable request.
